# Improving Electronic Sensor Reliability by Robust Outlier Screening

**DOI:** 10.3390/s131013521

**Published:** 2013-10-09

**Authors:** Manuel J. Moreno-Lizaranzu, Federico Cuesta

**Affiliations:** 1 Freescale^®^ Semiconductor Inc., 6501 William Cannon Dr., Austin, TX 78735, USA; E-Mail: manuel.j.moreno@freescale.com; 2 Escuela Técnica Superior de Ingeniería, University of Seville, Camino de los Descubrimientos s/n, Seville E-41092, Spain

**Keywords:** semiconductor device testing, zero defect, customer quality incident, robust statistics

## Abstract

Electronic sensors are widely used in different application areas, and in some of them, such as automotive or medical equipment, they must perform with an extremely low defect rate. Increasing reliability is paramount. Outlier detection algorithms are a key component in screening latent defects and decreasing the number of customer quality incidents (CQIs). This paper focuses on new spatial algorithms (Good Die in a Bad Cluster with Statistical Bins (GDBC SB) and Bad Bin in a Bad Cluster (BBBC)) and an advanced outlier screening method, called Robust Dynamic Part Averaging Testing (RDPAT), as well as two practical improvements, which significantly enhance existing algorithms. Those methods have been used in production in Freescale^®^ Semiconductor probe factories around the world for several years. Moreover, a study was conducted with production data of 289,080 dice with 26 CQIs to determine and compare the efficiency and effectiveness of all these algorithms in identifying CQIs.

## 1. Introduction

**Figure 1.**
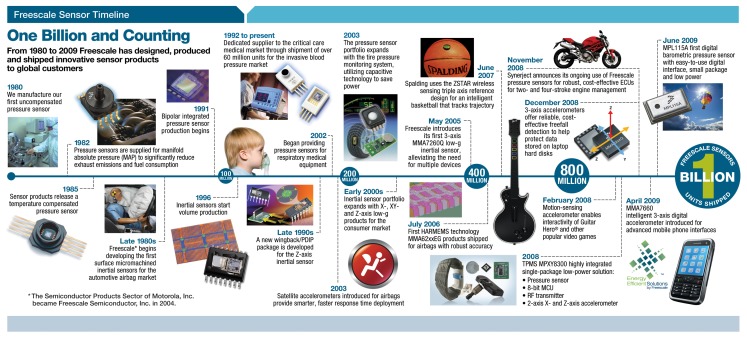
More than 30 years of Freescale Sensors.

Micro-electromechanical system (MEMS)-based sensors and actuators are nowadays widely present in electronic devices, ranging from cell phones, automotive components or medical equipment [1–3]. Indeed, Freescale^®^ Semiconductor has been developing sensors for almost 30 years (see Figure 1).

Semiconductor manufacturing is a very complex problem, which can be seen as being divided into four steps: wafer fabrication, probe, assembly and final test. The first step is wafer fabrication, in which integrated circuits (ICs) are fabricated layer by layer on silicon wafers. The next step is probe, where electrical tests are performed on each IC on the wafer to determine whether or not they are defective. Assembly follows next, in which non-defective ICs are enclosed into a package. Finally, in a final test, the packaged ICs go through an additional series of tests to filter out any possible defects added during the packaging operation. In spite of passing all the above tests, some devices contain latent defects that will manifest later in their life and that will originate a customer quality incident (CQI).

Sensor and actuator reliability is crucial in some application areas [4–6]. For instance, automotive electrical modules must perform below 10 defects per million (DPM) [7]. Since each module comprises hundreds of components, each one must have a DPM below one, which is virtually zero defects. A key factor for achieving zero defects is outlier detection methods. According to [7], these methods have varying degrees of efficiency, but can significantly improve quality. The benefit varies by device, technology and implementation method.

Spatial algorithms are a family of outlier detection methods based on the fact that defects tend to cluster. In [8,9], an approach based on a die-level neighborhood predictive model to successfully screen latent defects is proposed. By combining data mining with a defect-cluster extraction schema, it has been observed from production data that failing dice with traceable causes tend to form clusters at the wafer level or hot spots at the wafer-lot level. For semiconductor wafers, defect clusters appear as areas of random defect patterns at the wafer level, which are the result of random perturbations in the manufacturing process (particle related). Local defect patterns at the wafer-lot level are mostly located at a specific location and are process related.

Similarly, [10] arrived at the conclusion that some defects on wafers are not randomly distributed, but tend to cluster, based on burn-in data from 77,000 microprocessors manufactured at IBM. Furthermore, latent defects (CQIs) are normally found to cluster, with killer defects detected during probe. A later paper from the same authors [11] analyzed the same set of data results and introduced a yield-reliability model based on the fact that defects on a wafer have a tendency to cluster. This study also shows that that conclusion can be utilized to group devices into sets of different reliability based on how many of their neighbors failed. Dice that pass all the probe tests, but belong to a region with many defective dice have a higher probability of latent defects. Similarly, [12] show a strong correlation between probe data and device reliability. In [13] is investigated the use of defect characteristic models as yield models to screen latent defects for wafers with defect clusters. They propose and utilize a yield mining framework that guides manufacturers in determining if their device has a spatial relationship between the probe defects and latent defects. In [14], a modeling methodology is presented to link yield and reliability defects in a unified model.

Part Averaging Testing (PAT) is another family of outlier detection methods that are mostly applied to automotive devices that need to be qualified and tested to meet very demanding requirements, which are described in the Automotive Electronics Council standard AEC Q-100 [15].

In [15], the authors discuss two methods included in the AEC Q-100 specifications, which are Dynamic Part Averaging Testing (DPAT) and Statistical Bin Analysis (SBA), which comprise the statistical evaluation of failed bins. Similarly, in [16], DPAT is applied in testing inertial micro-electromechanical system (MEMS) devices, which are used as sensors. They claim that fabrication variations and defects can be caught using DPAT. These parts are referred to as outliers, and it is thought that, even though they passed the test program specification limits, they represent a portion of the population that usually will fail in the future.

Manufacturers must use innovative statistical methods to comply with zero-defect product quality requirements [17,18]. In [19], test methodologies that target those requirements are addressed. Outlier detection, part average testing and neighborhood screening are a few examples of those statistical methods. It is accepted that if all devices are tested the same way, dice from low-yield wafers, and low-yield regions within wafers, will likely be defective devices.

The work described in this paper is part of the global initiatives implemented in Freescale^®^ to ensure that the number of defective devices shipped to customers is as low as possible. The research presented in this paper focuses on new spatial algorithms (Good Die in a Bad Cluster with Statistical Bins (GDBC SB) and Bad Bin in a Bad Cluster (BBBC)) and an advanced outlier screening method called Robust DPAT (RDPAT), as well as two practical improvements that significantly enhance PAT methods.

Those algorithms have been widely used in production on every probe floor in Freescale^®^ in Asia, Europe and North America over the last seven years. During that time period, Electronic Wafer Mapping (EWM) has processed data for over ten million wafers. The percentage of wafers running PAT algorithms is 48% (with 17% being RDPAT), significantly contributing to device quality. It should be remarked that not all devices need to run these outlier detection algorithms. Devices for non-critical applications do not require them. Spatial algorithms were applied to 71%.

To determine the effectiveness and efficiency of these algorithms, 289,080 dice have been analyzed (with 205,671 good dice, 83,409 defective dice and 26 CQIs). Such studies are extremely rare in the field, since tracking CQIs is costly.

This paper is organized as follows: Section 2 describes the process of building sensors from silicon, showing the more critical phases and tests performed along them. Section 3 describes a standard spatial algorithm (GDBC) and presents two new ones, together with a performance comparison dealing with CQI. Section 4 addresses existing outlier detection Part Averaging Testing (PAT) methods applied to automotive devices. Section 5 introduces the new Robust DPAT algorithm. Section 6 discusses two new variations that improve PAT algorithms, including a comparison with conventional PAT methods. Section 7 ends with conclusions.

## 2. Semiconductor Manufacturing: From Silicon to Sensors

**Figure 2.**
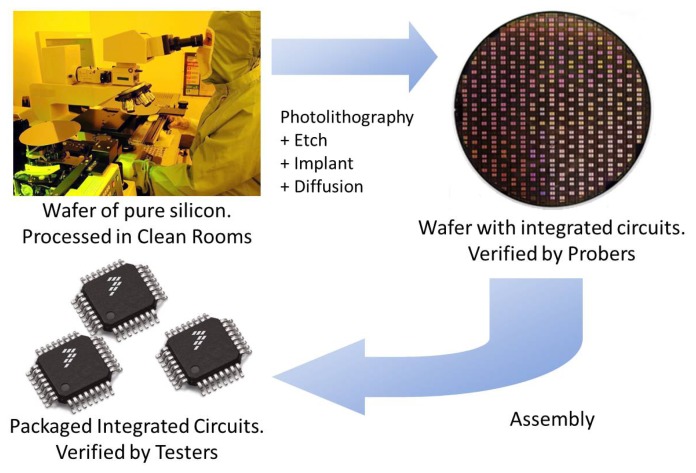
Semiconductor manufacturing steps.

Semiconductor manufacturing transforms silicon wafers into integrated circuits. Starting with wafers of pure, crystallized silicon (Figure 2), the processes described here build up a succession of layers of materials and geometries to produce thousands of electronic devices at microscopic sizes, which together function as integrated circuits (ICs). These processes require incredible precision and control.

Semiconductor manufacturing is divided into four major steps: wafer fabrication, probe, assembly and final testing. Those steps are explained next, as well as the main MEMS sensor manufacturing technologies.

### 2.1. Wafer Manufacturing

Integrated circuit (IC) designs are developed with Computer-Aided Design (CAD) systems. Designs are tested by simulation and perfected on computer systems before they are actually built.

ICs contain billions of components: transistors, resistors and capacitors, which are built on multiple layers, one on top of another. A glass photo mask is developed for each layer of the circuit, which will be used during photolithography (detailed later).

Silicon is the basic material of ICs. Turning silicon into ICs requires numerous steps and a lot of precision. The first step is to create the silicon wafers themselves. Then, multiple layers are built on the wafers to create the ICs, known as the wafer fabrication process [20]. Finally, a visual inspection is performed to detect particle contamination.

### 2.2. Crystal Growth and Wafer Slicing

The first step is the formation of a large silicon crystal (see [20] for an in-depth description). The silicon starts as granular powder that is melted. Then, a crystallized seed is dipped into molten silicon and, then, removed slowly as it rotates (Czochralski method). The result is a pure silicon cylinder called ingot. The diameter is either six (150 mm) or eight inches (200 mm).

Then, the silicon ingot is sliced into very thin wafers, which is done with a diamond saw. Each wafer is given a flat edge that will be used to orient the wafer correctly during later procedures. Finally, the wafers are polished until they are smooth and have the right thickness.

### 2.3. Wafer Fabrication Process

Semiconductor devices are fabricated in clean rooms to avoid particle contamination, which will damage the devices. Class 1 clean rooms are typical environments that restrict to no more than one particle of dust in a cubic foot of air. The air inside a clean room is filtered continuously, and operators wear special gowns and masks to keep the air particle-free.

Each single wafer will go through multiple steps to achieve the complex layers of conductor, semiconductor and insulating material needed. These steps are repeated dozens of times (once for each mask required by the circuit) to create the various layers necessary to build the circuitry.

The first layers deposited on the wafer create all the components, and the last layers connect these components. The following sections describe these steps.

#### 2.3.1. Photolithography

In this step, wafers are coated with photoresist, which is a light-sensitive substance. Then, a mask is used to expose portions of the wafer. This mask is carefully aligned, and ultraviolet light is applied. This light passes through the transparent sections of the mask and chemically modifies the photoresist on those areas. Lastly, a developer solution is applied to the entire wafer to remove the exposed photoresist. The non-exposed photoresist is left on the wafer, which will not react to etchants used in successive steps.

#### 2.3.2. Etching

The etching process follows photolithography to remove unwanted material from the wafer. This process removes oxide not protected by photoresist. This leaves a pattern on the wafer in the exact design of the mask. There are two main methods of etching: wet etching (using acids) and dry etching (using gas).

#### 2.3.3. Implant

The next step in the process consists of implanting ions (known as dopants) onto areas of the wafer that are not covered by the photoresist. These dopants are implanted just below the surface of the top layer and will modify the electrical characteristics of these selected areas, which encourage or discourage the flow of electrical current. Typical dopants are: boron, arsenic and phosphorous. After this step, wafers are heated in a process called annealing to reduce any possible damage incurred by the implant.

#### 2.3.4. Diffusion

Diffusion is performed in furnaces where an oxidation process occurs. Areas of the wafer not covered by the photoresist will be oxidized.

#### 2.3.5. Visual Inspections

The final step in wafer manufacturing is a visual inspection, where wafers are placed under a microscope and automatically scanned for particle contamination and structural defects.

### 2.4. Probe

At this point, all individual integrated circuits (also known as dice) are still on the wafer. During this step, these dice are tested for functional defects by applying special test patterns to them and reading the results.

Depending on the application for the device, testing is more or less aggressive. Devices for critical applications in the automotive and medical industries must comply with extremely low defect rates. Devices for those markets are tested more rigorously to ensure a small number of latent defects.

These electrical tests are conveyed on a piece of equipment called a prober (Figure 2). A set of microscopic contacts or probes, called a probe card, is positioned while the wafer is moved to make electrical contact with the probe heads. The software that determines what tests to apply and records the results is referred to as the test program.

There are two steps in the probe: Class probe and unit probe. In the class probe, an entire reticle of dice is tested. In the unit probe, an individual die is tested instead.

When a die (or array of dice) has been electrically tested, the prober moves to the next die (or array), and the next test is performed. The wafer prober is usually responsible for loading and unloading the wafers from their carrier (or cassette) and is equipped with automatic pattern recognition optics capable of aligning the wafer with sufficient accuracy to ensure correct registration between the contact pads on the wafer and the tips of the probes.

**Figure 3.**
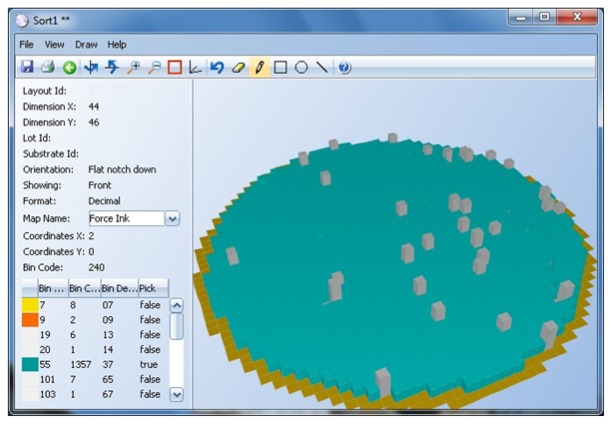
Hard bin 3D wafer view.

The results of these tests are measurements, like voltage, current, time delay, *etc.*, and are real numbers. These results are interpreted by the test program, and if any of the measurements are outside the specification limits, the die is considered defective. A bin number (integer) is assigned to the die to indicate if it passed (1) or if it failed (a number between 2 and 255, where each number indicates a different failure cause) (see Figure 3).

The proportion of dice on the wafer that have passed all the tests is referred to as the wafer yield.

In addition to these electrical tests, standard outlier detection algorithms are applied to devices for critical applications to further screen defective devices, as requested by clients in those demanding markets.

Finally, non-passing dice will be typically marked with a small dot of ink in the middle of the die (referred to as inking) before the next manufacturing step.

**Figure 4.**
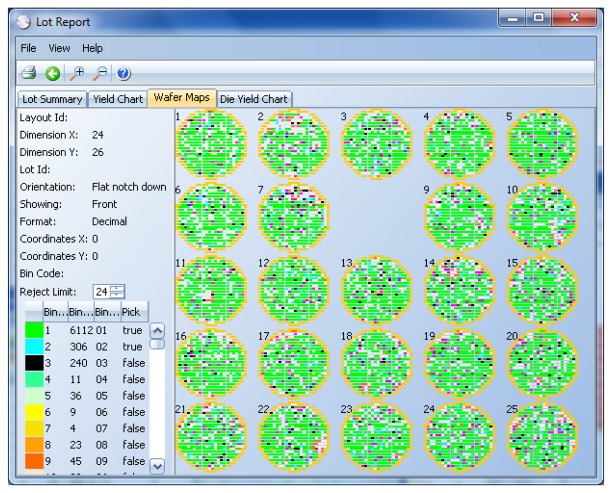
Wafer maps.

Alternatively, the information of passing/non-passing dice is stored in an electronic file, named a wafer map (see Figure 4). In this case, the process is referred to as inkless assembly. This map categorizes the passing and non-passing dice by making use of bins (1 for passing, 2 through 255 for failing). This wafer map is then sent to the assembly process, which only picks up the passing dice by selecting the bin number for good dice.

### 2.5. Assembly

At this manufacturing step, a diamond saw cuts the wafer into individual dice. The ones marked as defective during the probe step are discarded.

Then, the die bonding process takes place, which connects the pads on the chip to the frames with gold wires to create the electrical path between the chip and the package legs.

Finally, dice are encapsulated into plastic packages. Molten plastic is pressed around each die to form its individual package (Figure 2).

### 2.6. Final Test

Finally, additional tests are performed, which push chips to their extreme limits of performance to ensure a high quality, a reliable die and to assist engineering with product and process improvements.

During this final step in the manufacturing process, each chip is tested at various conditions to make sure the chip is still performing according to specifications. These conditions include cold, room and hot temperatures and, for some devices, a rigorous test, called burn-in, where the chips are placed in ovens at high temperature while electrical tests are applied to ensure reliability. Tests are performed by equipment known as testers (Figure 2). A device called a handler picks up devices to be tested, feeds them to the tester and discards the ones deemed as defective.

Finally, chips are inspected, sealed, labeled and shipped to customers.

### 2.7. MEMS-Based Sensor Technologies

There are two main technologies for sensor manufacturing: Bulk micromachining and surface micromachining.

#### 2.7.1. Bulk Micromachining

Bulk micromachining consists of creating the MEMS devices inside the silicon wafer by etching. In this way, the silicon is specifically removed in a subtractive process.

The main advantages of MEMS created by bulk micromachining are that they are made of a very stable mechanical material (the same silicon), and they can be manufactured in high volume with a cost reduction.

For instance, in the case of a piezoresistive pressure sensor, the silicon wafer is etched to form a diaphragm. This wafer is bonded with another one to form a vacuum-sealed cavity below the diaphragm that can deflect in response to the applied pressure. Pressure can be indirectly measured as a voltage based on the piezoresistive effect as a transduction method. Another example can be found in [21], where the design, fabrication and testing of a bulk micromachined inertial measurement unit is presented.

#### 2.7.2. Surface Micromachining

On the other hand, in surface micromachining, instead of etching the silicon wafer, the MEMS sensors are formed on top of it.

To that end, thin films of structural materials are deposited on top of the wafer. Some layers are deposited and partially removed to create gaps that can be used as a capacitor. Thus, an electrical signal can be obtained, based on the capacitive transduction method, when the gap changes due to external pressure.

## 3. Spatial Algorithms for Outlier Detection

Spatial Algorithms for outlier detection take advantage of the fact that defects tend to cluster. In this section, the standard Good Die in a Bad Cluster (GDBC) is recalled. After that, two new proposed methods are presented. Moreover, a comparison of those algorithms is performed.

### 3.1. GDBC

GDBC stands for Good Die in a Bad Cluster. Electric test results mark dice as good (all tests were passed) or bad (one or more test failed). This algorithm marks a good die as bad when it is surrounded by many bad dice.

The parameter for this algorithm is a threshold that indicates the percentage of bad neighbors needed to mark a good die as defective. If the device is for a critical application, this parameter will be set lower (more aggressive setting).

The rationale behind this method is that defects are normally clustered: A good dice surrounded by many defective ones has a high probability of containing latent defects. The higher the percentage of defective neighbors, the higher the probability that the device will fail in the future [11].

**Figure 5.**
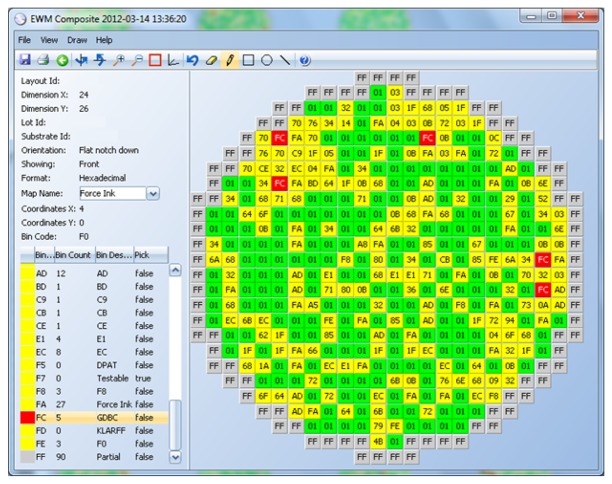
Good Die in a Bad Cluster (GDBC) example.

A GDBC example is shown in Figure 5 with an 87.5% threshold, meaning that a good die is marked as defective if 87.5% of its immediate neighbors or more are defective. Good dice are represented in green and bad, in yellow. The GDBC algorithm identified five dice (in red) as bad dice, which were almost completely surrounded by bad dice.

### 3.2. GDBC SB

A novel variation of GDBC named GDBC SB (Good Die in a Bad Cluster with Specific Bins) has been created, which only considers bad bins as a specific set of defective bins. The parameters for this new algorithm are the threshold of bad neighbors and the list of bins that are considered defective. The rationale behind this method is that some defects (defined by the bin number) are more likely than others to affect adjacent dice.

The list of defective bins included in this algorithm are determined by analyzing past CQIs and calculating the probability that a given bin number has an adjacent CQI. Bins with the highest probability will be included in the list. All the known CQIs should be included in this analysis. In the absence of CQIs, all defective bins are included, which defaults to the classic GDBC method. The performance of this method increases significantly with respect to the classic GDBC, as shown below.

### 3.3. BBBC

BBBC stands for Bad Bin in a Bad Cluster. This new algorithm identifies clusters of bad dice and, then, marks all good dice surrounding the cluster as bad. The rationale behind this method is the fact that certain clusters of defects have a high correlation to latent defects in neighboring dice. Similar to GDBC SB, the set of bins that make up the clusters are chosen based on past CQIs. If there are no CQIs, defect causes (bin numbers) that are suspected to spread to neighboring dice are used.

**Figure 6.**
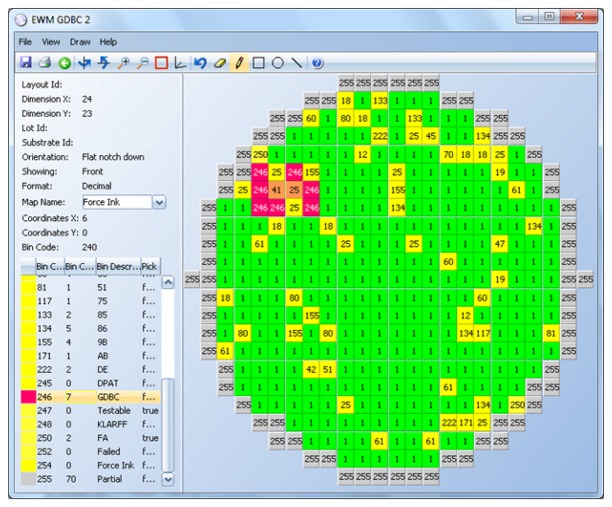
Bad Bin in a Bad Cluster (BBBC) example.

A BBBC example is displayed in Figure 6 Good dice are represented in green and bad, in yellow. Clusters of bins 25 and 41 are identified first (orange color). Only bins 25 and 41 that are surrounded by enough of bins 25 or 41 are considered part of a cluster. Then, all good dice surrounding the cluster are marked as bad (red color).

The parameters for this algorithm are:

- A list of bins that will be considered to form a cluster. In the example above, bins 25 and 41 only are used to determine clusters of bad dice.
- The threshold that indicates the percentage of bad neighbors that any of those bins need to have in order to form part of a cluster.
- Minimum cluster; if specified, only clusters that have the minimum number of dice specified by this parameter will be considered. If not specified, the minimum cluster size is one.

### 3.4. Spatial Algorithm Comparison

To determine the effectiveness and efficiency of these algorithms, 289,080 dice have been analyzed (with 205,671 good dice, 83,409 defective dice and 26 CQIs).

**Figure 7.**
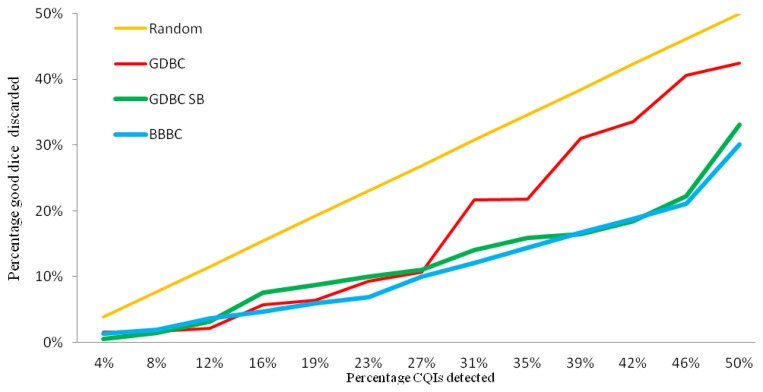
Spatial algorithms comparison.

Figure 7 compares the performance of the spatial algorithms: GDBC, GDBC SB and BBBC. The *X* axis shows the percentage of CQIs identified and the *Y* axis, the percentage of good dice discarded. The *X* and *Y* axes stop at 50%, since the allowed yield loss for outlier detection is much less than that.

**Figure 8.**
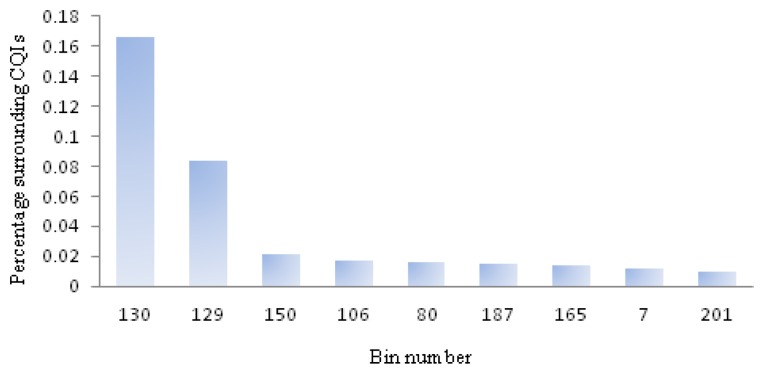
Bin to customer quality incident (CQI) correlation.

The orange straight line in Figure 7 represents the performance of randomly marking dice as a method of detecting outliers. If a percentage of the good dice were marked as outliers and not shipped to customers, the same percentage of CQIs would be detected, assuming defects are uniformly distributed on the wafers. For instance, to identify 48% of the CQIs would require discarding 48% of the good dice. Although this is a simplistic assumption, it provides a benchmark for other methods. The red line is the standard GDBC algorithm, which would discard 21.8% of dice to identify 34.6% of the CQIs. The GDBC SB, green line, in order detect 34.6% of the CQIs, would require a 15.9% yield loss, which is a significant improvement over GDBC. The bad bins included in this last GDBC version are determined by analyzing past CQIs and calculating the probably that a given bin number has an adjacent CQI. Figure 8 shows this distribution, where the *X* axis represents bin numbers and the *Y* axis, the probability that the given bin number has an adjacent CQI, considering all the good neighbors for all the known CQIs.

This probability is calculated as the number of CQIs around the given bin number divided by the total number of good dice that surround that bin number. All the known CQIs should be included in this calculation. In the absence of CQIs, all bad bins are included, which defaults to the classic GDBC algorithm. Finally, the other new spatial algorithm, BBBC, blue line, has a slight performance increase compared to GDBC SB: it would discard 14.4% of dice to identify 34.6% of the CQIs.

## 4. Part Averaging Testing (PAT) Outlier Detection

Part Averaging Testing (PAT) is a family of outlier detection methods that is mostly applied to automotive devices that need to be qualified and tested to meet very demanding requirements, which are described in the Automotive Electronics Council standard AEC Q-100 [15].

These algorithms are: Static Part Averaging Testing (SPAT), Dynamic Part Averaging Testing (DPAT), Automotive Electronics Council Dynamic Part Averaging Testing (AEC DPAT) and Nearest Neighbor Residual (NNR). All of them have been integrated into a Freescale^®^ application called Electronic Wafer Mapping (EWM) for testing wafers at probe. In the final test, SPAT and DPAT are usually considered.

Parametric test results need to be collected for each die to be able to run SPAT, DPAT, AEC DPAT and NNR. Hundreds of tests are typically executed.

In the following, these standard methods are described:

### 4.1. SPAT

SPAT stands for Static Part Average Testing. For each parametric test, lower and upper limits are determined to screen out dice that have a test result outside these specification limits. A series of electrical tests are performed and fed to EWM, which will determine if the test result for each die is within the limits for that test. Any die with one or more test results outside the test limits is marked as defective.

The parameters for SPAT are a list of tests and associated lower and upper limits.

### 4.2. DPAT

DPAT stands for Dynamic Part Average Testing. Unlike SPAT, limits are calculated for each wafer and test dynamically. Outliers are determined according to Equation (1), which is applied to each test individually, where the mean and standard deviation (stddev) are computed for the values for that test on the entire wafer (excluding results for defective devices), and *k* (sigma multiplier) is the parameter given for each test. Values outside the limits are considered outliers. The *k* multiplier is normally set to six (outliers are considered if outside six times the standard deviation from the mean). The smaller parameter *k* is, the smaller the upper and lower limits and the more dice that will be marked as defective.

(1) $$\begin{array}{c} \text{Upper Limit}=\text{mean}+k\times \text{stddev}\\ \text{Lower Limit}=\text{mean}-k\times \text{stddev} \end{array}$$

**Figure 9.**
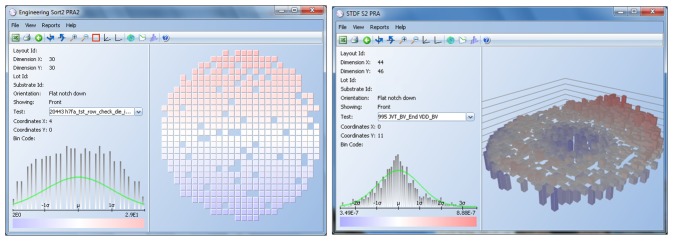
Dynamic Part Average Testing (DPAT) examples 2D and 3D wafer view.

Figure 9 shows an example of a parametric test for a wafer. Each die has a value assigned, which is a real number, and it is the result of the electrical test for that die. Wafer maps with parametric test results use a gradient from dark blue to dark red to show each value in relation to the mean of the test results for that wafer. The further the value is to the left of the mean, the darker the blue color; the further to the right, the darker the red color.

The rationale behind DPAT is that test results too far away from the mean are suspicious. The further away from the mean it is, the higher the probability of that die to fail in the future [15].

The parameters for DPAT are a list of tests and the associated sigma multiplier (*k*).

### 4.3. AEC DPAT

Another version of DPAT is known as AEC DPAT, which stands for Automotive Electronics Council Dynamic Part Average Test [15]. For each test, upper and lower limits are calculated, including all the values for non-defective dice for a wafer, as shown in Equation (2), where *p*1 and *p*99 are the first and 99th percentiles.

(2) $$\begin{array}{l} \text{Upper Limit}=\text{median}+k\times (p99-\text{median})\times 0.43\\ \text{Lower Limit}=\text{median}-k\times (\text{median}-p1)\times 0.43 \end{array}$$

This algorithm is, in general, more robust than DPAT, since it discards possible outliers from the dispersion calculation and includes certain correction for skewness in the distribution, increasing the efficiency. By avoiding the first and 99th percentiles, possible outliers are discarded from the limits calculation, increasing the statistical robustness. Additionally, by using a lower and upper limit, distribution asymmetry is factored in.

The parameters for AEC DPAT are a list of tests and the associated sigma multiplier (*k*). As mentioned above, the *k* multiplier is used to be more or less aggressive with the test, in particular.

### 4.4. NNR

**Figure 10.**
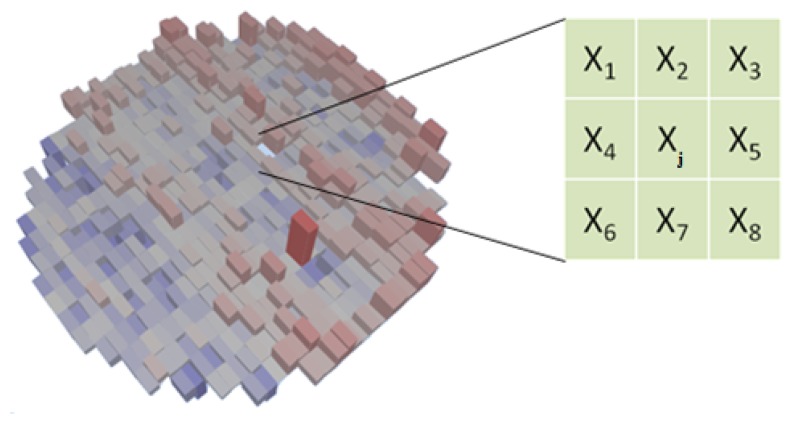
Nearest Neighbor Residual.

Another algorithm implemented is Nearest Neighbor Residual (NNR) [19]. The average test result of a die neighborhood (expected value) is subtracted from the die test result (measured value), as shown in Figure 10 and Equation (3). That residual is a real number and is then used to apply DPAT. This algorithm is effective when test results follow a gradient on the wafer, as depicted in Figure 9. Results at the top of the wafer are greater (red color) than results at the bottom (blue). The darker the red is, the further to the right of the mean, and the darker the blue, the further to the left of the mean it is. This algorithm highlights values that are significantly above or below the average values of their neighborhood. Engineers analyze test results and apply NNR to tests that display that type of gradient instead of the classic DPAT algorithm.

(3) $$\begin{array}{l} {\text{Residual}}_{j}={\text{Measured}}_{j}-{\text{Expected}}_{j}\\ {\text{Expected}}_{j}=\frac{\underset{j\neq i}{\text{∑}}w_{i}X_{i}}{\underset{j\neq i}{\text{∑}}w_{i}}\\ w_{i}=\exp\;\left[-\frac{{\left(\text{die}X_{i}-\text{die}X_{j}\right)}^{2}+{\left(\text{die}Y_{i}-\text{die}Y_{j}\right)}^{2}}{2\lambda^{2}}\right] \end{array}$$

The parameters for NNR are:

- Lambda (λ) determines the number of adjacent dice to be included in the neighborhood value calculation, as shown in Equation (3). The higher λ is, the more dice will be included in the neighborhood. Typical λ values are in the 1.5 to 2.0 range.
- List of tests and associated sigma multiplier, as defined in DPAT

## 5. Robust DPAT

A variation of DPAT, called R DPAT (Robust DPAT), has been developed and implemented to increase the efficiency and effectiveness of the DPAT algorithm. By using robust statistics (Grubbs algorithm), possible outliers do not bias the dispersion estimation. Additionally, by transforming non-normal data to normal, skewness is factored in (which is better than the AEC DPAT method).

Robust DPAT main steps are:

1. First, if the distribution has less than *γ* categories, an interpolation is performed to increase the granularity and raise the chances of passing the normality test and the Johnson transformation [22] being successful (if needed). A value of *γ* = 8 has been determined experimentally.
2. The next step is to test the distribution for normality (applying the Anderson-Darling test [23]).
3. If the distribution is normal, the Grubbs algorithm [24] is applied, which recursively removes outliers, and the algorithm ends. Grubbs is only valid if the underlining distribution is normal.
4. If the distribution is not normal, Grubbs is still applied to eliminate outliers. If the resulting distribution without the outliers is normal, then Grubbs was rightfully applied, since the underlying population is truly normal, and the process ends.
5. If the resulting distribution without the outliers is not normal, Grubbs is discarded, and a Johnson transformation is applied.
6. If the transformed distribution is normal, Grubbs is applied, and the algorithm ends.
7. If the transformed distribution is not normal, the AEC DPAT version (described in Section 4.2) is applied to the original data instead.

The Anderson-Darling normality test, Grubbs algorithm and Johnson transformation are described in the following subsections:

### 5.1. Anderson-Darling Normality Test

The Anderson-Darling normality test [23] is used as part of the robust DPAT algorithm. This test applies the cumulative density function to sorted values, with *y*_1_ being the lowest, *n*, the sample size and *F* being the theoretical cumulative value of the normal distribution, as shown in Equation (4).

(4) $$A^{2}=-n-\frac{1}{n}\sum_{i=1}^{n}\left(2i-1)(\ln F(y_{i})+\ln(1-F(y_{n+1-i}))\right)$$

The Anderson-Darling Test statistic, *A*^2^ [23], is adjusted for low sample sizes, and the *p*-value can be approximated using Equation (5). If the *p*-value is less than 0.05, the normality hypothesis is rejected.

(5) $$\begin{array}{l} {\hat{A}}^{2}=A^{2}(1+\frac{0.75}{n}+\frac{2.25}{n^{2}})\\ \begin{array}{cc} \left[{\hat{A}}^{2}<0.2\right] & \;p=1-e^{\left(-13.463+101.14{\hat{A}}^{2}-223.73{\left({\hat{A}}^{2}\right)}^{2}\right)} \end{array}\\ \begin{array}{cc} \left[0.2\leq {\hat{A}}^{2}<0.34\right] & p=1-e^{\left(-8.318+42.796{\hat{A}}^{2}-59.938{\left({\hat{A}}^{2}\right)}^{2}\right)} \end{array}\\ \begin{array}{cc} \left[0.34\leq {\hat{A}}^{2}<0.6\right] & p=e^{\left(0.9177-4.279{\hat{A}}^{2}+1.38{\left({\hat{A}}^{2}\right)}^{2}\right)} \end{array}\\ \begin{array}{cc} \left[0.6\leq {\hat{A}}^{2}<13\right] & \;p=e^{\left(1.2937-5.709{\hat{A}}^{2}+0.0186{\left({\hat{A}}^{2}\right)}^{2}\right)} \end{array}\\ \begin{array}{cc} \left[13\leq {\hat{A}}^{2}\right] & \;p=0 \end{array} \end{array}$$

### 5.2. Grubbs Algorithm

The robust DPAT algorithm uses the Grubbs Method [24] to identify outliers, but is only applicable if the underlying distribution follows a normal distribution. Grubbs does not use the standard deviation to identify outliers. It is instead an incremental algorithm that uses a distance from the average to detect outliers. The steps for the Grubbs algorithm as defined in [24] are:

1. Compute the *G_critical_* value, as shown in Equation (6), where *N* is the data sample size, *t* is the inverse Student's *t* cumulative distribution function and *α* is the level of significance or type I error, which is set at 0.05.
(6) $$G_{\text{critical}}=\frac{N-1}{\sqrt{N}}\sqrt{\frac{t^{2}(\frac{\alpha }{2N},N-2)}{N-2+t^{2}(\frac{\alpha }{2N},N-2)}}$$
2. Loop through all the values in the sample and compute their *G* value as (value - mean)/standard deviation, and get the maximum *G* value.
3. If the maximum *G* value is greater than *G_critical_*, mark that value as an outlier. Remove it from the sample. Go back to the first step to recalculate the *G_critical_* value, and continue removing outliers.
4. If the maximum *G* value is not greater than *G_critical_*, end the process.
5. Finally, Grubbs [24] only removes outliers temporarily, so that a robust mean and a robust standard deviation can be computed from the data values without outliers. Then, Equation (7) is applied to compute the upper and lower limits, where *k* is the sigma multiplier given for this algorithm. Data values outside these limits will be marked as outliers. By specifying the sigma multiplier (*k*), the algorithm can be tuned to be more or less aggressive.

(7) $$\begin{array}{c} \text{Upper Limit}=\text{robust mean}+k\times \text{robust stdev}\\ \text{Lower Limit}=\text{robust mean}-k\times \text{robust stdev} \end{array}$$

### 5.3. Johnson Transformation

The Johnson transformation [22] attempts to transform a non-normal data sample to normality. As described in [22], the first step is to generate 200 transformations and test them for normality. The one that yields the best results is chosen.

First, for each *z* ∈ {0.2, 0.21, 0.22….1.2}, the quantile ratio, *QR*, is calculated according to Equation (8), where *x_i_* is the *q_i_th* quantile for i =1,2,3,4 and *q*_1_, *q*_2_, *q*_3_, *q*_4_ are the areas on a standard normal curve below −3*z*, −*z*, *z* and 3*z*. Thus, *q*_1_ = Φ(−3*z*), *q*_2_ = Φ(−*z*), *q*_3_ = Φ(*z*), *q*_4_ = Φ(3*z*), where Φ is the distribution function of a standard normal variable.

(8) $$QR=\frac{\left(x_{4}-x_{3})(x_{2}-x_{1}\right)}{{\left(x_{3}-x_{2}\right)}^{2}}$$

If *QR* < 1, data values are transformed using functions *S_L_* and *S_B_*; otherwise, functions *S_U_* and *S_L_* are used. Functions *S_L_*, *S_B_*, *S_U_* are given in Equation (9). Parameters *η*, *γ*, λ and *ε* are estimated with Equation (10). These parameters must meet the conditions specified in Equation (9) or the transformation for that *z* value is discarded.

(9) $$\begin{array}{cccc} \text{Johnson Family} & \text{Transformation} & \text{Parameter Conditions} & X\;\text{Condition}\\ \begin{array}{c} S_{B}\\ \text{bound} \end{array} & Z=\gamma +\eta \ln\left(\frac{X-\varepsilon }{\lambda +\varepsilon -X}\right) & \begin{array}{c} \eta ,\lambda >0,\\ -\infty <\gamma <\infty ,\\ -\infty <\varepsilon <\infty \\ \eta >0, \end{array} & \varepsilon <X<\varepsilon +\lambda \\ \begin{array}{c} S_{L}\\ \text{lower bound or lognormal} \end{array} & Z=\gamma +\eta \ln(X-\varepsilon ) & \begin{array}{c} -\infty <\gamma <\infty ,\\ -\infty <\varepsilon <\infty \\ \eta ,\lambda >0, \end{array} & X>\varepsilon \\ \begin{array}{c} S_{U}\\ \text{unbound} \end{array} & Z=\gamma +\eta \sinh^{-1}\left(\frac{X-\varepsilon }{\lambda }\right) & \begin{array}{c} -\infty <\gamma <\infty ,\\ -\infty <\varepsilon <\infty \end{array} & -\infty <X<\infty \end{array}$$

(10) $$\begin{array}{ll} \text{Johnson Family} & \text{Parameters}\\ \begin{array}{c} S_{B}\\ \text{bound} \end{array} & \{\begin{array}{l} \hat{\eta }=\frac{z}{\cosh^{-1}\left(\frac{1}{2}{\left[\left(1+{\hat{x}}_{M}/{\hat{x}}_{U})(1+{\hat{x}}_{M}/{\hat{x}}_{L}\right)\right]}^{1/2}\right)}\\ \hat{\gamma }=\hat{n}\sinh^{-1}\left[\frac{({\hat{x}}_{M}/{\hat{x}}_{L}-{\hat{x}}_{M}/{\hat{x}}_{U}){\left[(1+{\hat{x}}_{M}/{\hat{x}}_{U})(1+{\hat{x}}_{M}/{\hat{x}}_{L})-4\right]}^{1/2}}{2({\hat{x}}_{M}^{2}/({\hat{x}}_{L}{\hat{x}}_{U})-1)}\right]\\ \hat{\lambda }=\frac{{\hat{x}}_{M}{\left[{\left\{(1+{\hat{x}}_{M}/{\hat{x}}_{U})(1+{\hat{x}}_{M}/{\hat{x}}_{L})-2\right\}}^{2}-4\right]}^{1/2}}{\left({\hat{x}}_{M}^{2}/{\hat{x}}_{L}{\hat{x}}_{U}-1\right)}\\ \hat{\varepsilon }=\frac{1}{2}\left({\hat{x}}_{2}+{\hat{x}}_{3}-\hat{\lambda }+\frac{{\hat{x}}_{M}({\hat{x}}_{M}/{\hat{x}}_{1}-{\hat{x}}_{M}/{\hat{x}}_{U})}{\left({\hat{x}}_{M}^{2}/({\hat{x}}_{L}{\hat{x}}_{U})-1\right)}\right) \end{array}\\ \begin{array}{c} S_{L}\\ \text{lower bound or lognormal} \end{array} & \{\begin{array}{l} \hat{\eta }=\frac{2z}{\ln({\hat{x}}_{U}/{\hat{x}}_{M})}\\ \hat{\gamma }=\hat{\eta }\ln\left[\frac{{\hat{x}}_{U}/{\hat{x}}_{M}-1}{{\left({\hat{x}}_{U}{\hat{x}}_{M}\right)}^{1/2}}\right]\\ \hat{\varepsilon }=\frac{1}{2}\left[{\hat{x}}_{2}+{\hat{x}}_{3}+{\hat{x}}_{M}\left(\frac{{\hat{x}}_{U}/{\hat{x}}_{M}+1}{{\hat{x}}_{U}/{\hat{x}}_{M}-1}\right)\right] \end{array}\\ \begin{array}{c} S_{U}\\ \text{unbound} \end{array} & \{\begin{array}{l} \hat{\eta }=\frac{2z}{\cosh^{-1}\left[\frac{1}{2}({\hat{x}}_{U}/{\hat{x}}_{M}+{\hat{x}}_{L}/{\hat{x}}_{M})\right]}\\ \hat{\gamma }=\hat{\eta }\sinh^{-1}\left[\frac{\left({\hat{x}}_{L}/{\hat{x}}_{M}-{\hat{x}}_{U}/{\hat{x}}_{M}\right)}{2{\left({\hat{x}}_{L}^{2}/{\hat{x}}_{M}^{2}-1\right)}^{1/2}}\right]\\ \hat{\lambda }=\frac{2{\hat{x}}_{M}{\left({\hat{x}}_{U}{\hat{x}}_{L}/{\hat{x}}_{M}^{2}-1\right)}^{1/2}}{({\hat{x}}_{U}/{\hat{x}}_{M}+{\hat{x}}_{L}/{\hat{x}}_{M}-2){\left({\hat{x}}_{U}/{\hat{x}}_{M}+{\hat{x}}_{L}/{\hat{x}}_{M}+2\right)}^{1/2}}\\ \hat{\varepsilon }=\frac{1}{2}\left({\hat{x}}_{2}+{\hat{x}}_{3}+\frac{{\hat{x}}_{M}({\hat{x}}_{L}/{\hat{x}}_{M}-{\hat{x}}_{U}/{\hat{x}}_{M})}{\left({\hat{x}}_{U}/{\hat{x}}_{M}+{\hat{x}}_{L}/{\hat{x}}_{M}-2\right)}\right) \end{array} \end{array}$$

**Figure 11.**
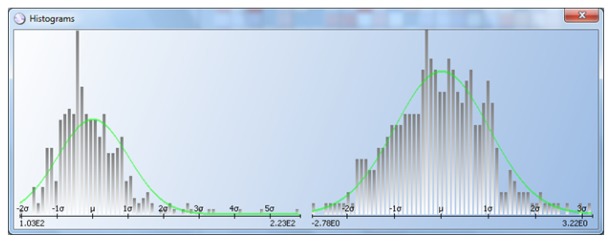
Log-normal to normal distribution transformation.

**Figure 12.**
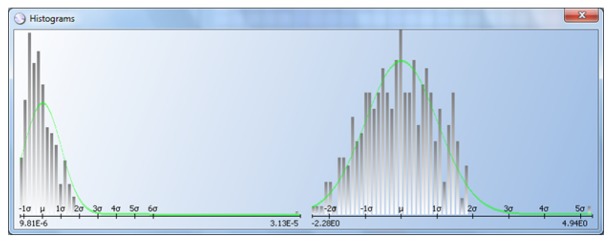
Exponential to normal distribution transformation.

Figure 11 shows a log-normal distribution (left) transformed to normal (right). Exponential distributions can also be transformed to normal, as shown in Figure 12. The data sample on the left follows an exponential distribution, and it is transformed to normal on the right. In cases like these, the R DPAT algorithm is more efficient than the classic and AEC DPAT versions.

## 6. Improving PAT Algorithms and Comparison

Results achieved by PAT algorithms and R DPAT can be also improved by taking advantage of the knowledge on the semiconductor manufacturing process. Namely, they are based on considering specific tests that have a high correlation with CQI and separating test results by test site.

### 6.1. Specific Test

In the classic DPAT algorithm, for instance, using tests with no correlation to CQIs deteriorates the efficiency of this method. A novel variation of this method has been developed that only includes tests that have a strong correlation with past CQIs. All known CQIs should be considered. In order to determine which tests will be used, device engineers utilize EWM by running the CQI and test correlation reports. In the absence of CQIs, a list of key tests recommended by the division is used.

The performance of DPAT with this enhancement, *i.e.*, DPAT with Specific Test (called DPAT ST) improves considerably, as shown below. The same applies for AEC DPAT ST, NNR ST or RDPAT ST. The reason behind this improvement is that some electrical measurements are more related to latent defects than others. By avoiding (or by being less rigorous) with tests that have a lower correlation with latent defects, the efficiency increases.

### 6.2. Multi-Site Test Results

**Figure 13.**
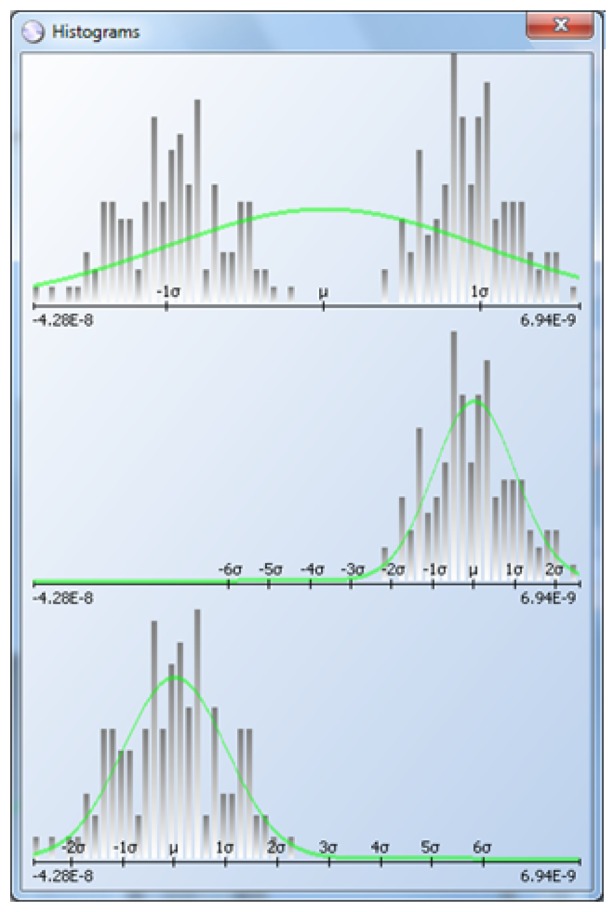
Multi-site example.

Some devices are probed with probe cards that have multiple probe heads (or sites): one, two, four, eight, up to 16. In some cases, the probe heads are not calibrated correctly and record results that are shifted with respect to one another. If all the results for a given wafer are included in the same histogram, the distribution will appear multi-modal and the classic DPAT algorithm will be less effective and efficient. The histogram at the top of Figure 13 shows such a case.

To avoid this problem, a multi-site option is added to all the different DPAT versions (DPAT, AEC DPAT and robust DPAT). If this multi-site option is selected, test results will be separated by site and will be treated independently before applying the desired DPAT version.

The histogram at the top on Figure 13 includes all the test results, which are clearly bimodal. The histogram at the center and bottom are the test results divided by site, and DPAT algorithms can then be applied to each one.

### 6.3. PAT Algorithms Comparison

To determine the effectiveness and efficiency of these algorithms, again, 289,080 dice have been analyzed (with 205,671 good dice, 83,409 defective dice and 26 CQIs).

**Figure 14.**
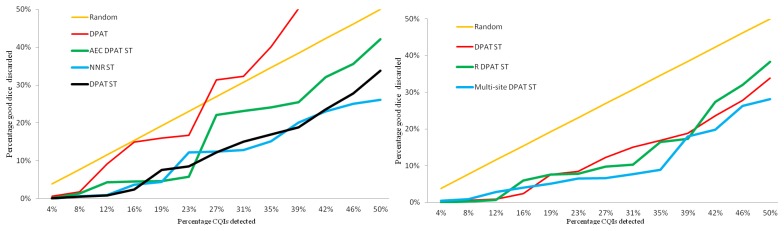
Part Average Testing algorithm comparison.

Figure 14 compares part averaging testing-based methods. The red line is the classic DPAT algorithm, including all the tests applied to this set of dice; approximately 1,500 tests. Using so many tests deteriorates this method's efficiency and effectiveness, since many of them have no correlation to CQIs. Identifying 34.6% of the CQIs (nine out of the 26 CQIs) would require discarding 40% of the good dice, which is worse than randomly discarding dice.

DPAT ST, black line, only includes tests that have a correlation with past CQIs. This information is gathered with the CQI and test correlation reports. By doing so, the performance improves considerably: 34.6% CQIs would be screened out, with just a 16.9% yield loss.

The green line shows the AEC DPAT enhanced by using ST. Its performance is slightly worse than DPAT ST: it would take discarding 24% of good dice to identify 34.6% of CQIs. The reason for this degradation is that the limits for each test are calculated with just the first, the 50th and the 99th percentiles, which do not include much detail about the distribution. Consequently, the performance decreases slightly with respect to the classic DPAT, which uses the mean and standard deviation to calculate the limits. On the other hand, enhanced NNR ST, blue line, represents a slight improvement over DPAT ST: to screen 34.6% of the CQIs, only 15.1% of the good dice would need to be discarded.

The other two new PAT algorithms that have been created are compared in Figure 14b. For the sake of clarity, just the DPAT ST algorithm, which can be considered the best among the ones shown in Figure 14a, has been included. With the R DPAT ST version, green line, performance has been improved: it only discards 16.5% good dice to screen 34.6% of CQIs.

Finally, the multi-site DPAT ST algorithm, blue line, significantly improves the performance: With just 8.9% yield loss, 34.6% of CQIs would be identified. The multi-site option splits results by probe site, eliminating the site to site variance in the outlier detection process, thus increasing performance.

## 7. Conclusions

Improving electronic sensor and actuator reliability in applications, like automotive devices or medical equipment, is paramount, since they should perform with an extremely low defect rate. Outlier detection algorithms are a key component in screening latent defects and decreasing the number of customer quality incidents (CQIs). This paper has presented two new spatial algorithms (GDBC SB and BBBC) and an advanced outlier screening method, called Robust DPAT (RDPAT), as well as two practical improvements that significantly enhance the existing algorithms. Those methods have been used in production in Freescale^®^ Semiconductor probe factories around the world for several years.

Moreover, a study was conducted with production data of 289,080 dice with 26 CQI to determine and compare the efficiency and effectiveness of all these algorithms in identifying CQIs.
